# Delayed Influence of Spinal Cord Injury on the Amino Acids of NO^•^ Metabolism in Rat Cerebral Cortex Is Attenuated by Thiamine

**DOI:** 10.3389/fmed.2017.00249

**Published:** 2018-01-15

**Authors:** Alexandra Boyko, Alexander Ksenofontov, Sergey Ryabov, Lyudmila Baratova, Anastasia Graf, Victoria Bunik

**Affiliations:** ^1^Faculty of Bioengineering and Bioinformatics, Lomonosov Moscow State University, Moscow, Russia; ^2^Belozersky Institute of Physico-Chemical Biology, Lomonosov Moscow State University, Moscow, Russia; ^3^Russian Cardiology Research-and-Production Complex, Ministry of Health of the Russian Federation, Moscow, Russia; ^4^Faculty of Biology, Lomonosov Moscow State University, Moscow, Russia; ^5^Faculty of Nano-, Bio-, Informational and Cognitive Technologies, Moscow Institute of Physics and Technology, Moscow, Russia

**Keywords:** amino acids metabolism, cerebral cortex, nitric oxide precursors, glutathione, spinal cord injury, thiamine

## Abstract

Severe spinal cord injuries (SCIs) result in chronic neuroinflammation in the brain, associated with the development of cognitive and behavioral impairments. Nitric oxide (NO^•^) is a gaseous messenger involved in neuronal signaling and inflammation, contributing to nitrosative stress under dysregulated production of reactive nitrogen species. In this work, biochemical changes induced in the cerebral cortex of rats 8 weeks after SCI are assessed by quantification of the levels of amino acids participating in the NO^•^ and glutathione metabolism. The contribution of the injury-induced neurodegeneration is revealed by comparison of the SCI- and laminectomy (LE)-subjected animals. Effects of the operative interventions are assessed by comparison of the operated (LE/SCI) and non-operated animals. Lower ratios of citrulline (Cit) to arginine (Arg) or Cit to ornithine and a more profound decrease in the ratio of lysine to glycine distinguish SCI animals from those after LE. The data suggest decreased NO^•^ production from both Arg and homoarginine in the cortex 8 weeks after SCI. Both LE and SCI groups show a strong decrease in the level of cortex glutathione. The neurotropic, anti-inflammatory, and antioxidant actions of thiamine (vitamin B1) prompted us to study the thiamine effects on the SCI-induced changes in the NO^•^ and glutathione metabolism. A thiamine injection (400 mg/kg intraperitoneally) within 24 h after SCI abrogates the changes in the cerebral cortex amino acids related to NO^•^. Thiamine-induced normalization of the brain glutathione levels after LE and SCI may involve increased supply of glutamate for glutathione biosynthesis. Thus, thiamine protects from sequelae of SCI on NO^•^-related amino acids and glutathione in cerebral cortex.

## Introduction

**Figure 1:**
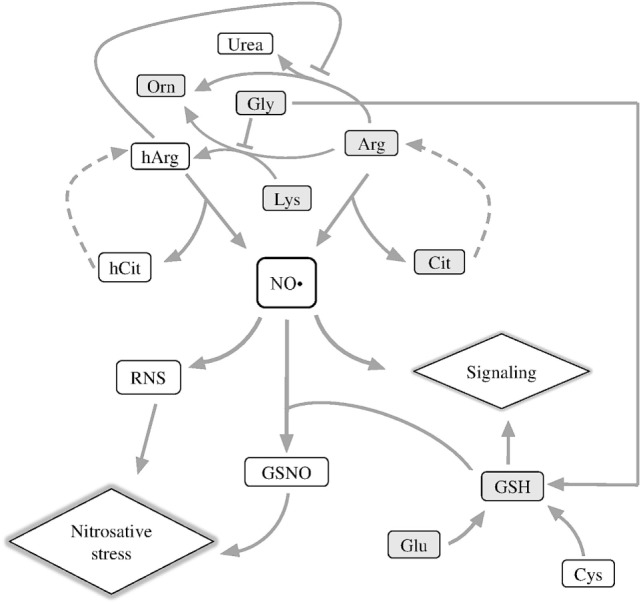
Schematic depiction of metabolic reactions associated with NO^•^ generation. Arg, arginine; Cit, citrulline; hArg, homoarginine; hCit, homocitrulline; GSNO, *S*-nitrosoglutathione; Lys, lysine; RNS, reactive nitrogen species; Orn, ornithine. The metabolites quantified in this work are shown in gray boxes.

In nervous tissues, nitric oxide (NO^•^), a gaseous messenger with multiple important roles in metabolism and homeostasis of animal and plant tissues, is shown to have both neurotoxic and neuroprotective effects depending on such factors as its local concentration (1) or the isoform of nitric oxide synthase (NOS; EC 1.14.13.39) producing this messenger (2, 3). Generation of NO^•^ is tightly linked to metabolism of both proteinogenic and non-proteinogenic amino acids, because NO^•^ precursors are arginine (Arg) or homoarginine (hArg) (Figure 1). hArg is synthesized from lysine (Lys) by Arg:glycine (Gly) amidinotransferase (EC 2.1.4.1). The enzyme is known to form guanidinoacetate and ornithine (Orn) from Gly and Arg (4). Lys is an alternative substrate of this reaction of Arg, leading to production of hArg instead of guanidinoacetate (Figure 1). Competition between the two acceptor substrates of Arg:Gly amidinotransferase is shown in Figure 1 as inhibition of the hArg synthesis from Lys by Gly.

Nitric oxide synthase oxidizes the imino group of the guanidine group of Arg or hArg, releasing NO^•^ and citrulline (Cit) or homocitrulline (hCit), correspondingly (Figure 1) (5, 6). In serum of patients with inflammatory myositis, the Cit level is a surrogate indicator of NO^•^ level (7). Cit and hCit are recycled to the precursors for NO^•^ production by argininosuccinate synthase and lyase (dashed lines, Figure 1) (8). Both Lys and hArg inhibit arginase, thus increasing the partition of the Arg flux to NO^•^ synthesis vs. urea cycle (9). As a result, NO^•^ generation required for NO^•^ signaling depends, in particular, on the proteinogenic amino acids Arg, Lys, Gly, and their non-proteinogenic derivatives hArg, Orn, Cit, and hCit (Figure 1, upper part).

Another important component of NO^•^ signaling, which couples NO^•^ to amino acids, is glutathione (GSH). The tripeptide has multiple biological roles, among others being a major component of cellular antioxidant system and a carrier and storage form of cysteine in the central nervous system (10). GSH is synthesized from Gly, glutamate (Glu), and cysteine, of which Gly is a competing substrate for the synthesis of the NO^•^ precursor hArg (Figure 1), and Glu is a neurotransmitter leading to oxidative stress under excitotoxic conditions. Together with NO^•^, GSH forms *S*-nitrosoglutathione (GSNO), which transduces NO^•^ signaling and damage through S-nitrosylation of proteins. As the source of bioavailable NO^•^, GSNO modulates a wide range of biological processes—from histone acetylation (11) to HIF-1α stimulation, contributing to functional recovery in traumatic brain injury (12).

In the spinal cord injury (SCI), NO^•^ has a huge impact on secondary injury progression, provoking oxidative stress and increasing levels of proinflammatory cytokines (13). Recently, severe SCI has been found to induce chronic inflammation in the brain, causing cognitive dysfunctions and depressive behavior associated with neurodegeneration in the brain regions distant from the injury site (14, 15). Such effects may underlie a well-known increase in frequency of the brain-affecting neurodegenerative diseases after SCI. Indeed, widespread neuroinflammation and microglia activation are landmarks of neurodegeneration in the brain (16), and several studies have already shown that SCI promotes these processes, also disturbing neurogenesis (17–19). Nevertheless, molecular mechanisms of delayed alterations outside the area of injury have been poorly explored. The NO^•^-mediated interactions between Arg and other amino acids, shown in Figure 1, may contribute to such delayed alterations. For instance, when Glu accumulates at synapses, the Arg- and hArg-generated NO^•^ aggravate the Glu excitotoxicity, accelerating neurodegeneration (20). Neurological disorders are also known to arise in hyperargininemia (21, 22), i.e., the state when Arg is accumulated in tissues. Depressed function of serotonergic neurons, accumulated free radicals, increased nucleotide phosphate hydrolysis, and inhibition of the Na^+^/K^+^-ATPase activity are among contributors to the brain damage by Arg-derived guanidino compounds (21–23).

Based on the data considered above (14, 15), we suggest that perturbations in NO^•^ metabolism and signaling, which contribute to the SCI-induced inflammation in cerebral cortex, may be manifested in the levels of the amino acids and GSH involved (Figure 1). On the other hand, inflammation, oxidative stress, autophagy, and endoplasmic reticulum stress leading to neurodegeneration, are known to be induced by thiamine deficiency (24–27), and we have recently reported that a high dose of thiamine results in a long-term optimization of the amino acid metabolism (28), potentially contributing to beneficial influence of thiamine on brain pathologies (29). It is also known that thiamine exerts analgesic and anti-inflammatory effects in different rat models including nerve injuries (30–33). The aim of this work is to estimate long-term changes in the NO^•^-related compounds in the cerebral cortex after SCI, and their potential normalization by thiamine treatment.

## Materials and Methods

### Animal Experiments

#### Animals

Our studies were performed on female Sprague–Dawley rats weighing 250–300 g. The animals were kept in standard conditions with 12 h light and 12 h dark cycle in individual cages with free access to water and meal. Manipulations with rats were carried out in accordance with the European Convention for the Protection of Vertebrate Animals Used for Experimental and Other Scientific Purposes (Strasbourg, 1986 ETS No. 123, Strasbourg, 18 March 1986). The experimental protocols were approved by Bioethics Committees of Russian Cardiology Research-and-Production Complex and Lomonosov Moscow State University.

#### Model of Severe SCI

SCI was performed by the weight-drop method, which allows for a highly reproducible procedure (34). The animals were narcotized intraperitoneally with a mixture of 5% ketamine solution (100 mg/kg) and 2% xylazine solution (20 mg/kg). The narcosis was sufficient for the total time of operation, which did not exceed 45 min after the administration of anesthesia.

Laminectomy (LE) was carried out at the level of TIX vertebra without affecting the dura mater, after which the spinal cord was fixed with clips by the spinous processes of TVIII and TXI vertebrae. Contusion was inflicted by a metal rod (2 mm in diameter, 10 g) vertically dropped from the height of 25 mm. The tube guiding the metal rod was fixed in stereotaxis along the midline of the spinal cord. After contusion (SCI groups), or right after LE (LE groups), the muscles and skin were sutured in layers, and an antibacterial spray was applied.

After surgery, the rats were placed into the individual cages with heating pad. Previous protocols (35–37) were adapted to this study involving postoperative administration of thiamine, which is known for analgesic and anti-inflammatory effects (30–33). Within the first 3 days after the operation, antibiotic (gentamicin, 40 mg/kg) was injected subcutaneously (s.q.) once per day to rats. Postoperative care included administration of saline–glucose solution within the first 5 days [2–3 ml twice a day intraperitoneally (i.p.)] and visual inspection for skin irritation or ulceration. Manual massage of the abdominal wall was carried out twice a day to relieve the bladder during the first 5 days until reflex bladder emptying was established. On the third day after surgery, rats could move using front paws, drank, and got food freely.

The level of functional recovery of hind legs was estimated by testing the locomotor activity of animals in open field using Basso, Beattie, Bresnahan Locomotor Rating Scale (BBB scale) (38). To take into account the training-induced biochemical changes in the brain, animals from both the operated (LE and SCI) and non-operated groups were tested. At the end of experiment (8 weeks after surgery), SCI and LE groups did not show a statistically significant difference in weight, compared with other groups. At this time point, rats were killed by decapitation, and their cerebral cortexes were extracted for further analysis as described below.

#### Administration of Thiamine

Thiamine was injected once i.p. at a dose of 400 mg/kg within 24 h after the operation.

#### Overview of the Rat Groups Employed in the Study

The experimental groups of animals studied are described in Table 1.

**Table 1:** Experimental groups and number of animals used for experiments.

Group abbreviation	Group description	Total number of animals included in study	Number of animals in group at the end of the study	Survival coefficient (%)
Control	Intact rats	7	7	100

Control + Th	Intact rats administered with thiamine	6	6	100

LE	Rats after anesthesia and laminectomy (LE) administered with gentamicin during 3 days after surgery	5	5	100

LE + Th	Rats after anesthesia and LE, administered with thiamine within 24 h and gentamicin during 3 days after surgery	6	6	100

SCI	Rats after anesthesia and contusion, admistered with gentamicin during 3 days after surgery	6	5	83

SCI + Th	Rats after anesthesia and contusion, administered with thiamine within 24 h and gentamicin during 3 days after surgery	6	5	83

### Preparation of Methanol–Acetic Acid Extracts of Rat Brain Tissue

After the rats were sacrificed by decapitation, the brain was taken out, put on ice, and the cerebral hemispheres (further called the cortex) were rapidly separated. The tissue samples were frozen in liquid nitrogen and stored at −70°C for at least 2 weeks before the extraction. To analyze the amino acid profiles, the cerebral cortex of experimental animals was extracted with methanol and acetic acid according to a published procedure (39).

### Quantification of Amino Acids and GSH in Rat Brain Extracts

Before chromatography, the brain extracts were filtered through Vivaspin 500, MWCO 3000 (Sartorius, Germany). Amino acids and GSH were quantified according to Ref. (40) using an L-8800 amino acid analyzer (Hitachi Ltd., Japan). For high performance liquid chromatography, the 2622SC-PF ion-exchange column (Hitachi Ltd., P/N 855-4507, 4.6 mm × 60 mm) was eluted at a rate of 0.35 ml/min by step gradients of Li–citrate buffers and temperature (in the range 30–70°C). On-line post-column amino acid derivatization was used for quantitative assessment of amino acids in the eluate, which occurred on mixing of the eluate with ninhydrin reagent solution (Wako Pure Chemical Industries; P/N 298-69601) supplied by a separate pump (+136°C, rate 0.35 ml/min). The stained products of derivatization were detected at 570 nm. Multichrom 1.71a software (Ampersand Ltd., Russia) was used to quantify the peaks obtained after chromatographic separation of the extracts.

In addition, the spectrophotometric method for the assessment of free cellular thiols based on application of the Ellman’s reagent—5,5′-dithiobis-2-nitrobenzoic acid (DTNB)—was used for determination of free GSH in rat brain extracts, taking into account that GSH is the main thiol in a cell. An aqueous 6 mM DTNB solution was used. The reaction mixture contained 0.4 M Tris–HCl buffer, pH 8.7, and 0.15 mM DTNB solution. The reaction was initiated by addition of extract to the reaction mixture. The volume of added sample ensured linear dependence of optical density on the amount of the extract. Maximum absorption at 412 nm was achieved during dark incubation of the mixture at 25°C for 3–5 min. Measurements were conducted with a Tecan Sunrise microplate reader (Austria) using 0.2 ml of the reaction mixture. Background absorption was measured in the reaction mixture without extract addition; the absorbance of sulfhydryl groups was obtained by subtraction of the background absorption from the total absorbance of the reaction mixture. The obtained concentrations of GSH were calculated using molar extinction coefficient (13,700 M^–1^ cm^–1^) and are presented in micromoles per gram of fresh tissue (FW). The agreement between the GSH assays by DTNB and using high performance liquid chromatography followed by ninhydrine reaction in the brain extracts was shown (40), allowing one to pool the results of the different GSH quantifications.

### Statistical Analysis

Gaussian distribution of the data was confirmed using the Shapiro–Wilk and Kolmogorov–Smirnov normality tests. The data are reported as a mean ± SEM. Statistical differences between each pair of groups, as well as an interaction between trauma and thiamine factors, were assessed with two-way analysis of variance (ANOVA) and *post hoc* Tukey’s test in GraphPad Prism 6 (GraphPad Software, Inc., USA). Two-tailed *p*-values <0.05 were considered as statistically significant.

## Results

### Influence of Spinal Injuries on the Amino Acids of NO^•^ Metabolism in the Rat Cerebral Cortex

In this study, we compared the three groups of rats after a long-term experiment of 8 weeks. To serve as a control for operative interventions, performed in the LE and SCI groups, the non-operated animals underwent the follow-up testing along with the LE and SCI animals, as such testing could affect the NO^•^ metabolism. LE is a surgical procedure when a part of the vertebral bone is removed. SCI includes the LE procedure, added by damaging of spinal cord. Hence, SCI animals were compared with LE animals to reveal specific contribution of neural tissue damage to the sequelae of SCI. The value of locomotor activity of animals after LE on the BBB scale was not different from that in non-operated rats (BBB score 21) already 2 weeks after the operation. By contrast, even after 8 weeks, the recovery of locomotor activity in the SCI-subjected animals was characterized by BBB scores 5.6 ± 0.51 that is far below the control values.

**Figure 2:**
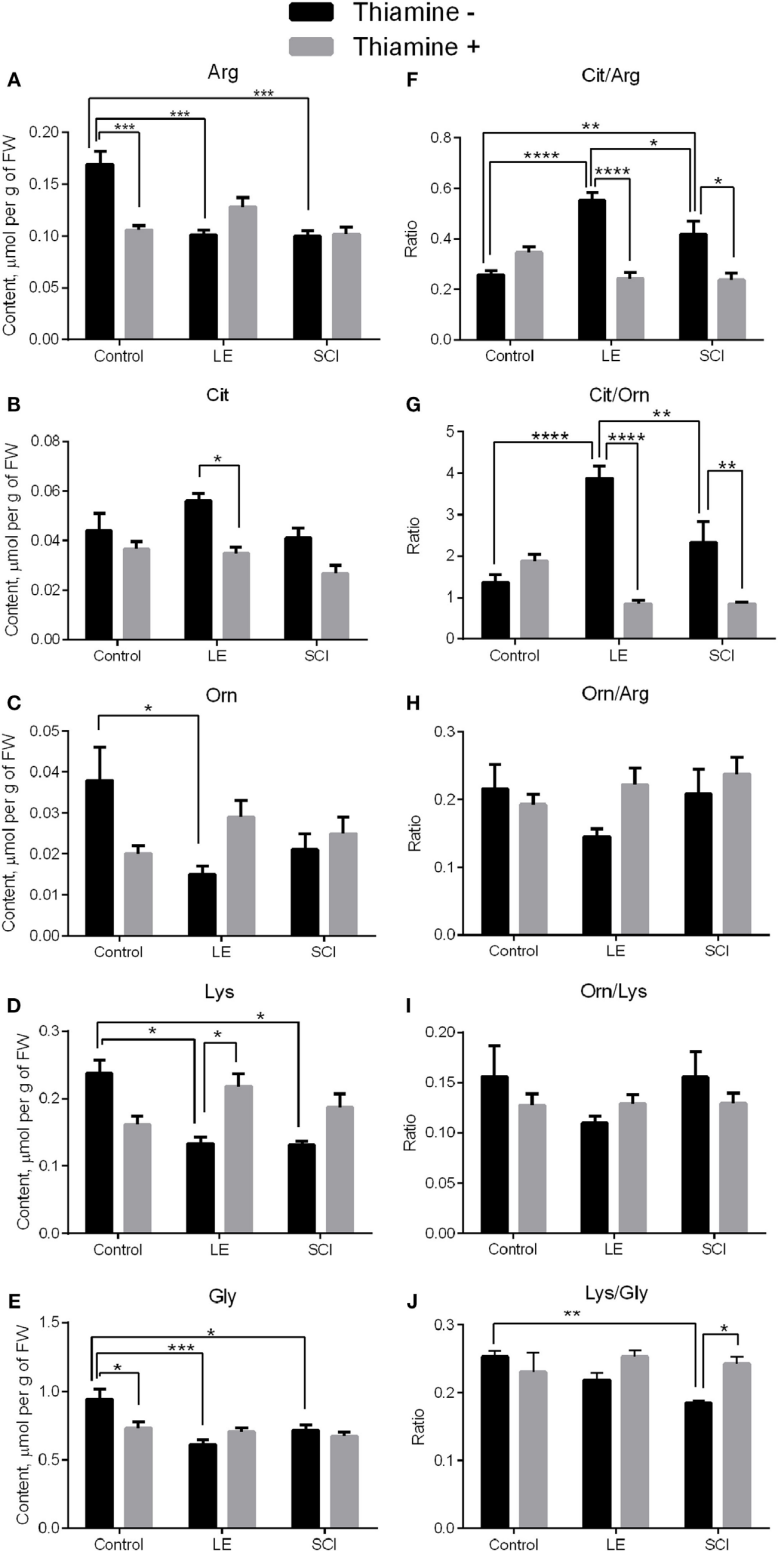
Changes in the levels of NO^•^-related amino acids in the rat cerebral cortex 8 weeks after spinal cord injury (SCI) or laminectomy (LE), with or without thiamine administration, in comparison with non-operated animals (control). **(A)** Arginine (Arg), **(B)** citrulline (Cit), **(C)** ornithine (Orn), **(D)** lysine (Lys), **(E)** glycine (Gly), **(F)** citrulline/arginine (Cit/Arg), **(G)** citrulline/ornithine (Cit/Orn), **(H)** ornithine/arginine (Orn/Arg) **(I)** ornithine/lysine (Orn/Lys), and **(J)** lysine/glycine (Lys/Gly)—the ratios between the NO^•^-related amino acids. Statistically significant differences between the groups are shown by asterisks only for each of the two factors: trauma or thiamine treatment. Complete statistical analysis is provided in Table 2. Number of asterisks is increasing with increased significance: *p < 0.05; **p < 0.01; ***p < 0.001; and ****p < 0.0001. Amino acid quantities are presented as micromoles per gram FW. Number of animals in each group is indicated in Table 1 (Section “Materials and Methods”).

Amino acids related to NO^•^ metabolism were investigated in rat cerebral cortex. According to Figures 2A–E (black bars), the levels of all amino acids except Cit decrease 8 weeks after LE compared with control rats. The levels of Arg, Lys, and Gly also fell down in rat cortex after SCI, contrary to non-operated animals (Figures 2A,D,E, black bars). No statistically significant differences between the LE and SCI groups are found for any of the amino acids under consideration (Figures 2A–E, black bars).

Apart from the mean level of metabolites, their distribution between different pathways may be changed. To characterize the changes in the distribution of Arg between the metabolic pathways presented in Figure 1, we characterized the partitioning by the amino acid ratios. According to Figure 1, the proportions of Cit to Arg, Orn to Arg, and Cit to Orn could serve as indicators of the relative involvement of Arg into the NO^•^ synthesis and urea cycle. As seen from Figures 2F,G, the Cit/Arg as well as Cit/Orn ratios increase after LE compared with non-operated rats. Cit/Arg is also higher after SCI, whereas Cit/Orn does not show statistically significant difference from the non-operated rats (Figures 2F,G, black bars). Importantly, the difference between the consequences of the two types of spinal injuries includes decrease of the Cit/Arg and Cit/Orn ratios in SCI compared with LE. Thus, the ratios of NO^•^-related amino acid are more sensitive indicators of the differences between LE and SCI, compared with the levels of amino acids as such. By contrast, Orn/Arg, which is the marker of urea cycle intensity, does not demonstrate any remarkable changes in the LE and SCI groups of rats in comparison with non-operated animals (Figure 2H, black bars).

The ratios of Orn to Lys (Orn/Lys) and Lys to Gly (Lys/Gly) could be used to estimate changes in the production of hArg by Arg:Gly amidinotransferase (Figure 1). Eight weeks after treatment, no statistical differences are shown for Orn/Lys between the groups under consideration, whereas the Lys/Gly ratio differs from non-operated rats in SCI animals, but not in those with LE (Figures 2I,J, black bars). Since Lys and Gly compete with each other for the common enzyme producing hArg in the reaction with Lys, the decrease in the Lys/Gly ratio in SCI animals suggests a less efficient hArg synthesis after SCI (Figure 1).

Thus, the levels of Arg and Lys in the rat cerebral cortex decrease in both the LE and SCI groups, compared with non-operated rats, suggesting changed metabolism of the amino acids due to operative interventions. At the same time, the ratios of Cit/Arg, Cit/Orn, and Lys/Gly, characterizing the involvement of Arg in the generation of NO^•^ and synthesis of hArg from Lys, are sensitive indicators which specifically address the consequences of the SCI-associated neurodegeneration for NO^•^ metabolism in the cerebral cortex of SCI animals, compared with LE where the spinal cord is not damaged.

### Influence of Thiamine Administration on the Amino Acids of NO^•^ Metabolism in the Rat Cerebral Cortex

A single high-dose thiamine injection within 24 h after SCI remarkably decreases the level of Arg and Gly in non-operated rats in comparison with those without thiamine (Figures 2A,E, gray bars), in accordance with our previous observation of the thiamine-induced long-term optimization of the amino acid metabolism in the rat cerebral cortex (28). At the same time, none of the amino acids under consideration in the LE and SCI groups with thiamine administration show any difference from the thiamine-treated non-operated rats (Figures 2A–E, gray bars). The finding points to the thiamine-induced normalization of the levels of the NO^•^-related amino acids in the cerebral cortex of the injured rats with or without neurodegeneration. The thiamine effect is further supported in the LE group, where the thiamine treatment decreases the level of Cit (Figure 2B, gray bars), simultaneously increasing that of Lys (Figure 2D, gray bars).

Regarding the amino acid ratios described earlier, the thiamine administration significantly decreases those of Cit/Arg and Cit/Orn in injured rats, bringing them to the same level as in the non-operated rats (Figures 2F,G, gray bars). The Orn/Arg and Orn/Lys ratios in all the thiamine-treated groups are maintained at the level of rats without thiamine (Figures 2H,I). Finally, the Lys/Gly ratio, which falls down in rats after SCI in comparison with non-operated animals, reliably increases to the control level in SCI with the thiamine administration (Figure 2J, gray bars). As a result, after the thiamine administration, the injured and non-operated groups are similar not only in the levels of the NO^•^-related amino acids (Figures 2A–E) but also in their ratios (Figures 2F–J), suggesting a positive effect of thiamine on the injury-induced perturbations in NO^•^ metabolism.

### Influence of SCI and Thiamine Administration on the GSH and Its Components Levels

**Figure 3:**
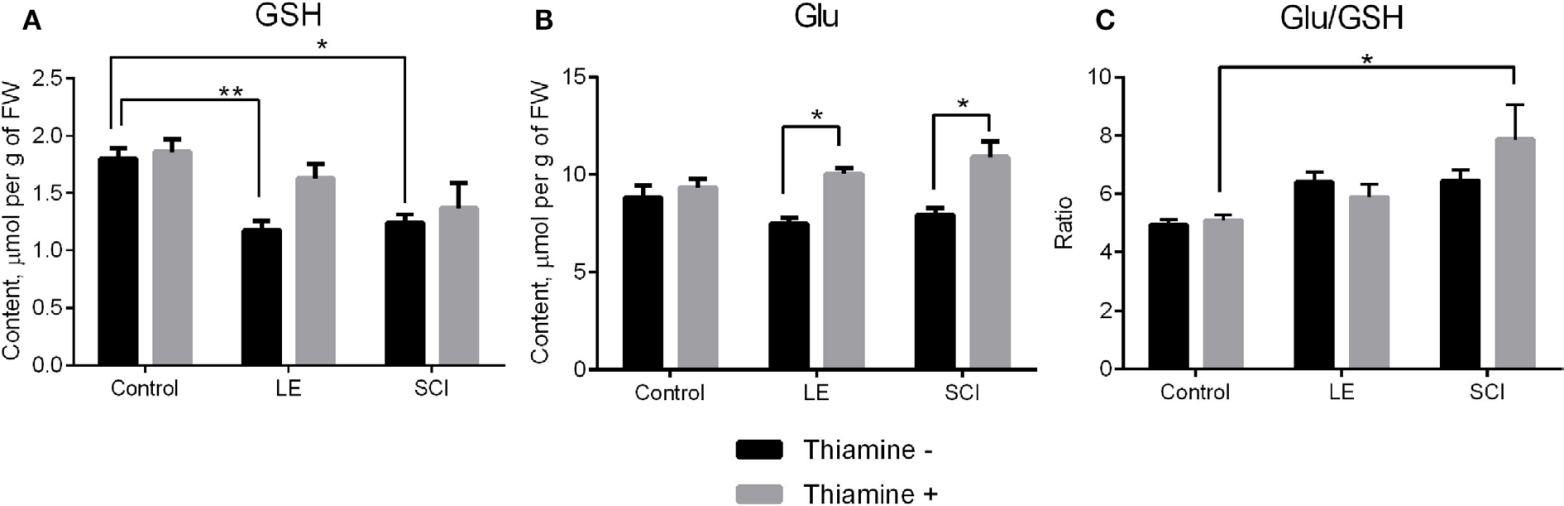
The glutathione (GSH) and glutamate (Glu) content of the rat cerebral cortex 8 weeks after spinal cord injury (SCI) or laminectomy (LE), with or without thiamine administration, in comparison with non-operated animals (control). **(A)** GSH, **(B)** Glu, and **(C)** the ratio glutamate/GSH (Glu/GSH). Other details are indicated as in the legend to Figure 2.

GSH, which is synthesized from Gly, cysteine, and Glu (41), is a major compound of cellular antioxidant defense, which is known to be impaired after SCI (42, 43). Our experiments show that the perturbations in the NO^•^-related amino acids in the cerebral cortex of rats 8 weeks after both LE and SCI are accompanied by significant decreases in the GSH level, compared with non-operated animals (Figure 3A, black bars). In particular, GSH is decreased along with a drop in its component amino acid, Gly (Figure 2E, black bars), although another component of GSH, Glu, does not change compared with control rats (Figure 3B, black bars). The thiamine injection normalizes the GSH content in the injured animals. As seen from Figure 3A (gray bars), the GSH decrease becomes insignificant in the thiamine-treated LE and SCI animals, compared with the non-operated control animals, whose GSH content is independent of thiamine. The normalization of the GSH levels by thiamine is accompanied by the thiamine-induced increases in Glu in injured animals compared with the non-operated thiamine-treated group (Figure 3B, gray bars). It is worth noting that either with or without the thiamine injection, the cerebral cortex Glu levels do not significantly differ between the LE, SCI and non-operated animal groups. The thiamine-induced increase in the Glu level in the LE and SCI animals (Figure 3B, gray bars vs. black bars) may contribute to the normalization of the GSH levels by thiamine (Figure 3A, gray bars vs. black bars), as the Glu–cysteine ligase reaction is rate limiting for GSH synthesis (44, 45). Although the cysteine availability is a crucial factor for this reaction (45), low cysteine content was not reliably detectable by our protocol of the amino acid analysis. Hence, we only estimated the ratio of Glu/GSH to assess the GSH production (Figure 3C). Compared with non-operated rats, both operations do not change the ratio (Figure 3C, black bars) when GSH is decreased in the absence of thiamine (Figure 3A, black bars). However, the injection of thiamine increases this ratio after SCI, compared with non-operated animals (Figure 3C, gray bars), which is accompanied by the normalization of the GSH levels in cerebral cortex of these animals (Figure 3A). The data suggest that the thiamine-induced elevation of Glu may stimulate the Glu–cysteine ligase reaction, thus contributing to the normalization of the GSH level under conditions where the GSH biosynthesis is impaired.

### Statistical Analysis of Influence of LE, SCI, and Thiamine Treatment on the Indicators of NO^•^ Metabolism and Oxidative Stress

Pairwise comparison of the specific groups of animals, allowing one to judge on the effects of spinal injuries of different severity (LE and SCI) and the protective effect of thiamine, are presented in Figures 2 and 3. The two-way ANOVA with *post hoc* Tukey’s test used for these estimations also provides additional information, which is given in Table 2. Statistical significance of the differences in all metabolites and ratios shown in Figures 2 and 3 are summarized in Table 2. In addition to the *p*-values for differences between the factor-determined group pairs (inside the cells of the table), the table provides *p*-values for the significance of the overall action of any factor, such as trauma or thiamine administration (*p*-value columns), and the interaction between the two factors (interaction *p*-value column), based on comparison of all the six experimental groups. According to Table 2, the factors of operation and thiamine treatment show significant interaction with each other, as judged from their influence on many studied amino acids and their ratios. When the levels of NO^•^-related amino acids Orn and Lys are considered, one may see no significant influence of each factor, but their strong interaction. According to other parameters, e.g., the levels of Arg and Gly, the interaction is revealed when only one of the factors shows significant influence. The most sensitive indicators associated with NO^•^ generation such as the ratios of Cit/Arg, Cit/Orn, and Lys/Gly demonstrate not only significant alterations by both injury and thiamine but also the significance of interaction between the two factors (Table 2). Thus, thiamine or trauma affects NO^•^ metabolism in cerebral cortex, dependent on the state of the animals. That is the thiamine effects are different in the normal and injured animals, as well as the trauma sequelae are different in the thiamine-treated and non-treated animals.

**Table 2:** Statistical analysis of the effects of trauma, i.e., operative interventions with [spinal cord injury (SCI)] or without [laminectomy (LE)] neuronal injury, and thiamine administration on the NO^•^- and GSH-related amino acids and GSH.

Indicator (compound or ratio)	Trauma factor							Thiamine treatment factor				Interaction between factors (*p*-value)

	*p*-Value	Control vs. LE		Control vs. SCI		LE vs. SCI		*p*-Value	Th− vs. Th+			
	
		Th−	Th+	Th−	Th+	Th−	Th+		Control	LE	SCI	
Arg	***0.002***	***4 × 10^−4^* ↓**	0.480	***3 × 10^−4^* ↓**	1.000	1.000	0.178	0.153	***6 × 10^−4^* ↓**	0.324	1.000	***1 × 10^−4^***
Cit	*0.055*	0.411	1.000	0.997	0.662	0.259	0.820	***7 × 10^−4^***	0.819	***0.031* ↓**	0.326	0.326
Orn	0.337	***0.049* ↓**	0.738	0.250	0.977	0.977	0.981	1.000	0.155	0.352	0.994	***0.01***
Lys	0.127	***0.013* ↓**	0.262	***0.011* ↓**	0.936	1.000	0.738	0.187	0.102 ↓	***0.036 ↑***	0.377	***5 × 10^−4^***
Glu	0.546	0.659	0.937	0.916	0.393	0.997	0.780	***2 × 10^−4^***	0.989	***0.032* ↑**	***0.014* ↑**	*0.098*
Gly	***0.001***	***3 × 10^−4^* ↓**	0.998	***0.023* ↓**	0.957	0.730	0.993	0.165	***0.040* ↓**	0.678	0.989	***0.008***
GSH	***1 × 10^−4^***	***0.007* ↓**	0.752	***0.020* ↓**	*0.074***↓**	0.999	0.674	***0.040***	0.999	0.122	0.983	0.238
Cit/Arg	***0.005***	***1 × 10^−4^ ↑***	0.105	***0.003 ↑***	*0.107*	***0.035* ↓**	1.000	***1 × 10^−4^***	0.183	***1 × 10^−4^* ↓**	***0.002* ↓**	***1 × 10^−4^***
Orn/Arg	0.414	0.595	0.968	1.000	*0.865*	0.768	0.997	0.252	0.993	0.400	0.981	0.238
Cit/Orn	***0.006***	***1 × 10^−4^ ↑***	*0.056*↓	*0.085* ↑	*0.076* ↓	***0.004* ↓**	1.000	***1 × 10^−4^***	0.625	***1 × 10^−4^* ↓**	***0.006* ↓**	***1 × 10^−4^***
Orn/Lys	0.327	0.496	1.000	1.000	1.000	0.593	1.000	0.401	0.851	0.966	0.898	0.312
Lys/Gly	*0.080*	0.437	0.767	***0.010* ↓**	*0.982*	0.571	0.977	***0.031***	0.822	0.342	***0.028 ↑***	***0.009***
Glu/GSH	***0.001***	0.354	0.865	0.315	***0.010 ↑***	1.000	0.121	0.413	1.000	0.983	0.475	0.195

*Cit, citrulline; Orn, ornithine; Glu, glutamate; Arg, arginine, Lys, lysine; Gly, glycine*.

*The direction of changes of the parameters in the pairwise comparisons of the groups is indicated by the upwards or downwards arrows next to the *p*-values for *p* ≤ 0.1. Significant differences (*p* ≤ 0.05) are highlighted in bold italics. Trends (*p* ≤ 0.1) are shown in italics. *p*-Values for significances of each factor and for the factor interactions are provided in the corresponding columns. *p*-Values for the pairwise comparison of animal groups are written inside the corresponding cells*.

## Discussion

### Delayed Consequences of LE and SCI in the Rat Cerebral Cortex

SCIs are known to result in chronic inflammatory response in cerebral cortex, leading to neurodegeneration and functional impairment (15, 18), but the molecular mechanisms of such perturbations remain to be unraveled. The role of NO^•^ signaling in the short-term progression of acute inflammation was extensively studied, with enhanced NO^•^ synthesis having both positive and negative effects during inflammatory process (46). To understand potential contribution of the NO^•^-related pathways into the sequelae of SCI in areas of nervous system distant from the damage site, we performed quantification of the brain amino acids involved in NO^•^ metabolism (Figure 1). Multiple changes in their levels that occur in the cerebral cortex of rats after either LE or SCI (Figure 2) demonstrate a high impact of not only the injury of spinal cord (SCI group) but also the associated operative intervention (LE group) on the NO^•^-related amino acids of the rat cerebral cortex.

Our work reveal that the ratios of Cit to Arg or Orn, which estimate the Arg partitioning between the NO^•^ synthesis and urea cycle (Figure 1), are much more sensitive markers of the alterations occurring in the NO^•^-dependent pathways of operated animals, than the levels of amino acids. The Cit/Arg ratio increases under pathological conditions, i.e., in both the LE and SCI animals, compared with non-operated rats (Figure 2F). Similar changes occur with the Cit/Orn ratio, although this indicator is less sensitive. For instance, unlike the Cit/Arg ratio, that of Cit to Orn does not show statistically significant difference between the SCI group and non-operated animals (Figure 2G). Given the fact that Cit has been shown to serve as an indicator of tissue NO^•^ generation (7), the elevation of Cit/Arg together with stable Cit level under significantly decreased Arg (Figures 2A,B,F) could be interpreted as an increase in NO^•^ production after both LE and SCI, according to Figure 1. Enhanced NO^•^ synthesis is also indirectly suggested by the fact that the Orn/Arg ratio, which estimates the Arg partitioning into the urea cycle (Figure 1), does not change after SCI and LE, as compared with non-operated animals (Figure 2H).

Remarkably, the Cit/Arg and Cit/Orn ratios are significantly lower in SCI group than in LE group (Figures 2F,H), suggesting NO^•^ synthesis to be impaired after SCI. The impairment may result from intensive NO^•^ formation in the acute phase of SCI, which may exhaust the NO^•^ sources and/or lead to NO^•^-dependent damage by the time of our analysis, i.e., 8 weeks after the injury. This is further supported by the SCI-specific decrease of the Lys/Gly ratio vs. non-operated rats, which is not seen in the LE group (Figure 2J). The lower Lys/Gly ratio points to a stronger competition of Gly with Lys for the enzyme generating hArg (Figure 1) in the brain of SCI animals. The ensuing decrease in the biosynthesis of additional NO^•^ source, hArg, may also contribute to the impairment in the NO^•^ generation in the cerebral cortex of SCI rats.

Interestingly, in the cohort study of Alzheimer’s disease influence on Arg metabolism (47), changes of NO^•^-dependent pathways are similar to those in our study. In particular, cerebral cortex in Alzheimer’s disease is characterized by a constant level of Cit and decreased ratio Cit/Arg. Thus, the NO^•^-related indicators in the neurodegenerative processes in the Alzheimer’s disease cortex, where also total NOS activity is shown to be reduced (47), are similar to those which we see in the cerebral cortex of rats subjected to SCI. The similarity is consistent with the impaired synthesis of NO^•^ in the cerebral cortex of SCI rats, characteristic of neurodegenerative changes as delayed consequences of SCI in the brain. The similarity also suggests that the observed changes in the rat brain biochemistry stem from neurodegenerative processes in CNS rather than surgery and associated effects.

The GSH content is decreased after both operations (Figure 3A), consistent with impairment of the amino acid metabolism, contributed by nitrosative stress (Figure 1) as a result of operative intervention with or without neurotrauma. GSNO (Figure 1) is a main source and transporter of NO^•^ radical, thus participating in NO^•^-dependent regulation and damage. The GSH decrease observed in the LE and SCI animals may thus reflect nitrosative stress occurring as a chronic consequence of spinal injuries. At the same time, since GSH in the brain is considered as a carrier and storage form of cysteine (10), its decrease in operated animals suggests disturbed cysteine metabolism in the rat cerebral cortex.

### Delayed Consequences of Thiamine Administration in the Rat Cerebral Cortex

Our previously published data show that a high-dose thiamine (vitamin B1) injection employed in this study has a long-term effect on the amino acid metabolism. In rat cerebral cortex, it decreases levels of many amino acids, with Arg and Gly among them (Figures 2A,E). The finding suggests an adaptive response, sparing amino acids from increased degradation after activation of the ThDP-dependent dehydrogenases due to the thiamine administration (28). Remarkably, in the non-operated rats, the adaptive response does not affect the amino acid ratios, confirming the maintenance of the normal metabolic state in the thiamine-exposed cerebral cortex, despite the homeostatic decreases in the amino acid levels. However, in operated rats, thiamine influences the amino acid levels in such a way, that the critical parameters (Cit/Arg and Cit/Orn) that reveal the differences between the LE and SCI groups in the absence of thiamine, become not different from the control level in the presence of thiamine (Figures 2F,G). Thus, the important long-term effect of the thiamine post-SCI administration is the normalization of metabolic indicators of NO^•^ generation in the rat cerebral cortex during chronic phase of SCI.

Figure 3B suggests that the positive effect of thiamine on the NO^•^-dependent pathways in SCI could be due to protection of the thiamine diphosphate-dependent 2-oxoglutarate dehydrogenase complex. The complex regulates metabolic flux through tricarboxylic acid cycle where amino acids are degraded (48) and is known to be impaired in neurodegeneration (49). Decreased mitochondrial respiration shown in the rat model of traumatic brain injury (50) may be due to the 2-oxoglutarate dehydrogenase complex impairment under these conditions. This has been confirmed in a recent study, also establishing the thiamine-dependent protection of the 2-oxoglutarate dehydrogenase activity and mitochondrial respiration of Glu in traumatic brain injury (51). It is known that inhibition of 2-oxoglutarate dehydrogenase complex in rat cerebral cortex decreases the Glu level (52, 53). Our results on the thiamine-induced increase in Glu after LE and SCI (Figure 3B) may thus be related to the normalized Glu metabolism when the 2-oxoglutarate dehydrogenase complex, known to be impaired in neuropathologies including traumatic brain injury (51), is protected.

The increase in the cerebral cortex Glu, caused by thiamine injection, may contribute to the thiamine-induced alleviation in the decrease of brain GSH (Figure 3A). As shown in Figure 3C, thiamine significantly increases the Glu to GSH ratio in SCI animals compared with non-operated ones. Although this is consistent with incomplete normalization of the GSH biosynthesis by thiamine, increased Glu may be used not only for the Glu–cysteine ligase reaction, which is rate limiting for GSH synthesis (44, 45) but also for the Glu exchange for cysteine (54), increasing intracellular availability of the other reaction substrate, cysteine, which could not be reliably quantified in our study. Remarkably, the Glu to GSH ratio is increased by thiamine administration only in SCI group, remaining unchanged under normal physiological conditions and in LE group (Figure 3C). Nevertheless, no statistically significant interaction between the effects of trauma and thiamine on this ratio could be revealed (Table 2).

By contrast, statistical analysis reveals a strong interaction between the thiamine administration and trauma factors regarding NO^•^ metabolism (Table 2). The profound protective effect of thiamine is demonstrated by the lack of differences between the operated and non-operated rats after the thiamine treatment (Table 2; Figure 2, gray bars).

Thus, from comparison of the three groups of rats studied in this work (non-operated, after LE and after SCI), we can conclude that the cerebral cortex of operated animals strongly differs from that of non-operated ones, regarding the NO^•^-related amino acids and GSH levels 8 weeks after the operations. Deleterious effects of SCI, compared with LE, on NO^•^ signaling in the cerebral cortex are manifested in the decreased ratios of Cit to Arg and Cit to Orn. As a result, our data indicate perturbed system of NO^•^ production and signaling, induced in cerebral cortex as a chronic SCI-associated pathology. A single high dose of thiamine within 24 h after SCI normalizes the delayed perturbations in the NO^•^-related amino acids and GSH in the cerebral cortex, induced by SCI. This finding is in good accordance with the efficacy of high-dose thiamine in Alzheimer’s disease (29), where similar changes in the NO^•^-related amino acids of cerebral cortex are shown to be associated with the reduced activities of total NOS (47).

## Ethics Statement

This study was carried out in accordance with the European Convention for the Protection of Vertebrate Animals Used for Experimental and Other Scientific Purposes (Strasbourg, 1986 ETS No. 123, Strasbourg, 18 March 1986). The experimental protocols were approved by Bioethics Committees of Russian Cardiology Research-and-Production Complex and Lomonosov Moscow State University.

## Author Contributions

All coauthors contributed substantially to the conception or acquisition and/or analysis of the data and drafting the work. All coauthors have provided final approval of the version to be published, as well as agree to be accountable for all aspects of the work.

## Conflict of Interest Statement

The authors declare that the research was conducted in the absence of any commercial or financial relationships that could be construed as a potential conflict of interest.
